# Mistakes in Thinking about Cognitive Science and How to Reduce Them

**DOI:** 10.1523/ENEURO.0380-24.2024

**Published:** 2024-10-04

**Authors:** P. N. Johnson-Laird

**Affiliations:** ^1^Department of Psychology, Princeton University, Princeton, New Jersey 08540; ^2^ Visiting Scholar, Department of Psychology, New York University, New York, New York 10003

**Keywords:** fallacies, mistakes, reasoning, thinking

## Abstract

This article allows readers to assess their ability to detect errors in thinking in seven case histories of psychologists’ thoughts about cognitive science. It explains the nature of the errors and shows that some of them reflect faulty reasoning. It presents a “model method” to improve reasoning. It is based on the theory of mental models, which gives a general account of how individuals think, both deductively and indicatively, and which postulates that individuals construct mental models of possibilities in the world. The model method enhances both the accuracy and speed of reasoning. The article concludes with some general reflections on the role of knowledge of meanings, the world, and context in thinking.

## Significance Statement

This article shows that cognitive scientists sometimes make mistakes in thinking. It illustrates seven actual mistakes. Some of them are a result of erroneous reasoning, and the paper presents a simple method that in experiments shows improvement in both the accuracy and speed of reasoning. Other errors also reflect a lack of pertinent knowledge.

From Wundt onward, we all can make mistakes in thinking about cognitive science. The aim of this article is to convince you of this point and to describe a method to help you to improve your thinking. Mistakes from ignorance seem more excusable than those from faulty thinking. Aristotle's ignorance led him to claim that the heart is the organ of emotion and intellect. But faulty thinking seems a plausible cause of his conclusion that heavy objects fall faster than light objects. Nothing prevented him—except cognitive inertia—from dropping an iron and a wooden ball together from a height and observing their coincidental impact on the ground. Yet knowledge and thinking are hard to disentangle, and a mistake is not always a *mistake*. When a jazz musician improvises a wrong note, she may find a melodic continuation that makes it sound deliberate, thereby creating a new musical idea. A great computational theorist and a great physicist both defended the potential value of mistakes (Turing, 1996; Wilczek, 2002). But, like the jazz musician, it remains better to recognize a mistake than to let it slip by unnoticed. In fact, you don't need logic to do so, as you will discover if you read on.

Many books and articles have long lists of fallacies and inferential errors. There are also individual accounts of errors, and an admonitory catalog of them (Sternberg, 2019), but I have yet to find a comprehensive theory of mistakes in thinking. And a fallacy can be a mistake only because it violates a particular logic. Formal logics treat an inference of the following sort as fallacious:

The frequency of beta waves is greater than that of alpha waves, and the frequency of alpha waves is greater than that of theta waves.

Therefore, the frequency of beta waves is greater than that of theta waves.

Why is it a fallacy? Because no premise asserts that the relation of “greater than” is transitive and therefore warrants such an inference. But you know the meaning of “greater than”, and you can use it to construct a model of the relative frequencies of the three sorts of wave, from which you can draw the conclusion (you could build an actual physical model or a mental model that is abstract or that underlies an image). The inference is a fallacy only to someone who demands formal axioms (aka “meaning postulates”) to capture the logical properties of words that are not part of logic. Otherwise, meanings embodied in models do just as well. Humans were able to think before they created logics and axioms. These systems led to supreme intellectual achievements, but helping people to avoid errors in reasoning is not one of them.

Mistakes in scientific thinking tend to be subtle—scientists would hardly make them if they weren't. To set our agenda, I have listed seven cases of mistakes that cognitive and behavioral scientists made, and you might like to jot down what they were if you identify them. The information in my descriptions and your knowledge should suffice to do so.

1. A polymath, who knew Darwin, observed insects that he had trained to come to a source of food in his sitting room. Many experiments led him to conclude that they had difficulty in finding their way around. What mistake had he made?
2. If the first-born in a family is of exceptional intelligence, then the second-born is likely to be less intelligent. A researcher suggested that the omega-3 lipids in breast milk are known to benefit a child's intellect, but the mother's diet cannot provide it all, and so some of it comes from her bodily reserves. As a result, the first child gets more omega-3 than the second child. What mistake did this theorist make?
3. A cognitive scientist carried out an experiment to examine a set of inferences that varied in their complexity according to a particular logic. The participants judged whether the conclusion to each inference followed from the premises. The results, as the scientist conceded, were less than satisfactory. What three mistakes did he make?
4. Some theorists argue that the principles of reasoning are relative to each culture, and so one culture's rules are as good as those of any other culture. What mistake did the “relativists” propounding this theory make?
5. To assess “If A happened, then C happened,” you add A hypothetically to your beliefs, make a minimal adjustment to them to accommodate A if it conflicts with them, and then assess whether C happens. Several theorists have supported this theory. What mistake does it make as cognitive science?
6. A psycholinguist once defined an optimist as “anyone who believes that optimists exist.” People tend to accept this definition, because you'd hardly be an optimist if you didn't think that they exist. What mistake does the definition embody?
7. A psychologist carried out an experiment in which the task was to discover the rule governing triples of numbers, including this starting example: 2 4 6. The participants generated their own triples and received feedback about whether or not each of them was an instance of the rule. They tended to generate triples that were instances of their own hypotheses about the rule, which they had to write down too. The psychologist concluded that they had a “confirmation bias”—a tendency to seek evidence that confirms a given hypothesis. What mistake did the psychologist make?

Let's look first at the mistakes and then consider their origins.

1. Lubbock (1882) was a polymath who studied insects. He tested bees in these studies. His mistake was to put their target of honey only in his living room. Bees encode the direction of nectar from their hive in relation to the sun—even if they have to fly a dog-leg (von Frisch, 1993). Their famous “waggle” dance on a honey comb in the hive communicates both this direction in relation to the sun and the distance of the nectar. Lubbock, had he known, would have kicked himself: he could have made von Frisch's observations. His mistake was not to think of how hives would survive if bees couldn't find their way back to them, and at least to manipulate the location of the bee's food in his experiment so that in one condition it was ecologically sensible, i.e., outdoors. Darwin recognized that bees were able to fly the same routes again, and he did wonder how they could do it.
2. The fall off in intelligence from first- to second-born does not necessarily call for any causal explanation. It could be nothing more than regression to the mean. Exceptional individuals are much rarer than average individuals. So, given a first-born child of exceptional intelligence, the second-born child tends, by nothing more than chance, to be less intelligent, i.e., to regress toward the mean. It doesn't follow that the researcher's argument about omega-3 lipids is false, but it needs to take into account this explanation, see Don Davis's answer at https://www.quora.com/Question-That-Contains-Assumptions-Why-are-first-born-children-more-intelligent-than-second-born-children.
3. This experimental study made three mistakes (Osherson, 1976). It aimed to investigate reasoning, but the participants could instead merely have tried to guess whether the conclusion followed [guessing was reliably more prevalent in on-line reasoning experiments than in those carried out face to face (Ragni and Johnson-Laird, 2020)]. Psychologists often use the procedure of presenting a conclusion for participants to evaluate or a set of conclusions from which they have to select an optimal one—they did so for 70 years from the first published experiment to study reasoning in 1908. But it is not a good procedure for an initial study of a new domain of reasoning. The experimental design in the present case also erred in examining only inferences that are valid in the logic. So it was impossible to discover whether people make inferences that are not valid in the logic. Most people, for instance, given the information that it may rain, infer that it may not rain (if you reject this inference, you are concluding that it must rain in most modal logics), but this inference is not valid in the logic under investigation though Aristotle accepted it. The third mistake that the investigator made is one I plead guilty to. For many years, I assumed without question that logical validity is the appropriate criterion for assessing reasoning in daily life (more on this point, later).
4. Cultural relativists postulate that each culture's principles of reasoning are as good as any other cultures' despite their clashes on some inferences (Barnes and Bloor, 1982, and a multitude of postmodernists). There is a culture of rationalists and its members—include me in, please—who believe that there are universal criteria for correct reasoning. Suppose relativists allow that rationalists are right in their culture. It follows that the principles of reasoning are universal for all cultures, because that it is what rationalists believe. So relativism is wrong. Suppose instead that relativists reject this argument. Then they also reject that rationalists are right in their culture. It follows that there is a culture whose principles of reasoning are not right, and so relativism is wrong. Either way, it is wrong (Johnson-Laird, 2006, p. 275). Beware the horns of a dilemma!
5. A distinguished philosopher, Stalnaker (1968), proposed this semantics for conditionals, including those such as my claim: “If the Viennese had three legs, then they would march in waltz time.” Various psychologists have adopted this account, e.g., Evans and Over (2004). Philosophy need not concern itself with the practicality of its logics. But consider these two disjunctive beliefs:
  The pupil's swollen or it's dilated, but both are not true.
  It's swollen or it's not dilated, but both are not true.

Could both beliefs be true in a particular situation? Most people think so, which tells us something important about thinking, which I explain presently. The two beliefs are disjunctions constructed out of two fundamental propositions about the pupil, it's swollen and it's dilated, which could each be true or false, so they yield 2^2^ potential cases. The first of the two disjunctions rules out two of these four cases as false, namely:

*It's swollen and it's dilated*. (They are not both true.)

*It's not swollen and it's not dilated*. (One is true.)

The second of them rules out the other two cases as false:

*It's swollen and it's not dilated*. (They are not both true.)

*It's not swollen and it's dilated*. (One is true.)

So no possibility exists in which both the disjunctive beliefs are true, i.e., they are inconsistent with one another. Suppose you have a set of scientific beliefs constructed out of 100 fundamental propositions. To determine whether your beliefs are consistent, you need to check that at least one of the 2^100^ cases is true. This number is far too vast for a search of all the cases to be tractable, i.e., the number is over 30 digits long. Yet, if none of these cases is true, your beliefs are inconsistent (Goldreich, 2010). The psychologists’ mistake was to assume without question that the consistency of a set of beliefs can be checked in a tractable amount of time in daily life. Could some—as yet unknown—method condense beliefs, or their combinations, to allow a more efficient assessment of their consistency? No-one knows. So perhaps we are bound to live with the risk of inconsistent beliefs.

1. The problem with the definition of optimism is that it infects the world with too many instances of this happy state of mind. I believe that optimists exist and so I am an optimist; you now believe that I am an optimist, so you are an optimist, and so on and on. Soon, everyone is an optimist—even avowed pessimists. The definition spins an endless loop (Johnson-Laird, 2006, Ch. 11).
2. The investigator was mistaken to attribute his participants’ performance to confirmation bias (Wason, 1960), because a person can select an instance of a hypothesis just because it is an instance without any concern for the truth or falsity of the hypothesis (Nickerson, 1998). By the way, the correct rule in the experiment was as follows: any three numbers increasing in order of magnitude.

To understand mistakes, and to ameliorate them, we have to know something about how thinking occurs. The theory that my colleagues and I have developed over the last 50 years is easy to describe, but you will find the evidence corroborating it elsewhere; see https://www.modeltheory.org/. Thinking manipulates mental models of possibilities in the world, real or imaginary, which are constructed from perception and knowledge. Reasoning formulates conclusions from these models. Something in common lurks in all seven cases of mistaken thinking, and it seems to be the single biggest cause of human errors and human disasters. We overlook possibilities.

This neglect even underlies common fallacies. Consider this example inferred from a hypothesis and an empirical result:

*Hypothesis*: If working memory is for sounds then similar-sounding words will be harder to remember than dissimilar-sounding words.

*Result*: Similar-sounding words are harder to remember than dissimilar-sounding words.

*Conclusion*: Therefore, working memory is for sounds.

The conclusion may well be true, but the inference is an error. And it exemplifies the inferential problem at the heart of science. General scientific hypotheses are unprovable. To understand why, consider the hypothetical premise (1) above. It refers to three possibilities, whose abbreviations are presented here in three vertical columns, in which the word “not” denotes the negation of a proposition:

**Table:** 

Possibility 1	Possibility 2	Possibility 3
Memory is for sounds	Not (memory is for sounds)	Not (memory is for sounds)
Similar sounds are hard	Not (similar sounds are hard)	similar sounds are hard

If the hypothesis is true, its possibility (1) in the left column is that working memory is for sounds and similar sounds are hard to remember, its possibility (2) is that neither of these propositions holds, and its possibility (3) is that working memory is not for sounds, but similar sounds are hard to remember—for some other unstated reason. The second premise reports the true empirical result that similar sounds are hard to remember in working memory, and so we eliminate the contrary possibility (2), as shown by the red line crossed through it:

**Table:** 

Memory is for sounds	Not (memory is for sounds)	Not (memory is for sounds)
Similar sounds are hard	Not (similar sounds are hard)	Similar sounds are hard

When you have eliminated all which is impossible, as Sherlock Holmes remarked, then whatever remains, however improbable, must be the case. As you see, what remains are two possibilities (1) and (3), but in only (1) is working memory for sounds. In the other possibility, (3), working memory is not for sounds. So the inference to the conclusion above—that working memory is for sounds—does not follow, because possibility (3) is a counterexample to it. The error is well known as “affirming the consequence” and identified in Aristotle's *Sophistical refutations*. You make an inference from a corroboration of the consequence of a conditional, its then-clause, to conclude that its if-clause holds. Of course, if you could rule out possibility (3) too then the inference would follow. But any finite set of data is consistent with a countable infinity of explanations, and so you cannot eliminate all those that explain why similar sounds are harder to remember in the short term other than that working memory stores sounds.

A contrasting case is one in which the hypothesis is the same, but experiments show that similar sounds are not harder to remember than dissimilar ones. This discovery eliminates two of the hypotheses’ possibilities (1 and 3) and leaves only possibility (2), from which it follows:

Therefore, working memory is not for sounds.

Experiments cannot prove hypotheses, but in principle they can disprove them. Alas, theorists can often find wiggle room in the details of an experiment to allow them to continue to believe that their hypothesis is still viable, e.g., the experiments did not test enough participants to have the statistical power to detect a significant effect.

The use of diagrams in the way I illustrated above is known as the “model method” because Victoria Bell, who invented it when she was a graduate student working with me, based it on the theory of mental models as it applies to reasoning (Johnson-Laird, 2006, p. 288 et seq.). She showed that it takes ∼2 min to teach people the model method. Yet participants who then used it in an experiment were correct on 95% of inferences whereas the control participants were correct on a reliably smaller 63% of them. Likewise, those who used the method took a mean of ∼15 s for each inference, whereas the control group took a reliably longer mean of 24 s. The method worked even when participants were not allowed to use paper and pencil. Readers should know, however, that certain sorts of inference to which the model theory applies (Khemlani and Johnson-Laird, 2022) call for more powerful and as yet untested diagrams.

The model method shows that you do not need a formal logic in order to make inferences. You merely enumerate the possibilities that the premises describe and draw a conclusion from them. If the conclusion describes all the possibilities and nothing else then it follows as a necessary inference. If it describes only some of them and does not deny any of the others then it follows as a possible inference. Such a conclusion that is described as a possibility is a necessary inference, e.g., It is swollen or dilated; therefore, it is possible that it is swollen. A measure on set of models is basis for probabilities. You may now be thinking to yourself: doesn't the method merely implement a logic, not as formal rules, but as a semantics? Plausible, but not so. To see why, consider standard logics, such as the sentential calculus, which deals with logical idealizations of *not*, *and*, *if,* and *or,* and all the other logics of which it is a part. The criterion for a correct inference in these and other logics is validity, and an inference is valid if its conclusion is true in every case in which its premises are true (Jeffrey, 1981, p. 1). If this principle seems plausible to you, then you haven't grasped its full implications. Consider this inference:

The first-born is intelligent or the second-born is intelligent, but not both are.

Therefore, the first-born is intelligent or the second-born is intelligent, or both are.

Is this inference correct? Most people say, “no,” which accords with the model theory, because the conclusion describes a possibility—both first-born and second-born are intelligent—that is not one that the premise claims. Indeed, the premise rules it out. Nevertheless the inference is logically valid: in the two cases in which the premise is true, the conclusion is true. A corollary is that any set of premises yields infinitely many valid conclusions, e.g.:

The first-born is intelligent.

Therefore, the first-born is intelligent, or a hippo is in your bath, or both.

You may say that the conclusion is ridiculous. It is. But it is valid. As a result, no standard logic can identify which conclusion you should draw from a set of premises, because no logical principles can single out sensible conclusions from the infinitude of ridiculosities. Few people realize this point, and so it is worth repeating: if you can draw your own correct conclusion from a set of premises, you are doing something that is beyond any logic that relies on validity. The model method, however, enables you to draw your own sensible and necessary conclusions. Your reasoning transcends logic.

Earlier I referred to mistakes resulting from ignorance and those resulting from faulty thinking. Most inferences depend on knowledge of meaning, knowledge of context, and knowledge of the world. Consider this:

The rule concerns even numbers alone or numbers of any sort.

In fact, it concerns numbers of any sort.

Therefore, it does not concern even numbers alone.

The inference looks similar in kind to the fallacious example about working memory. But it is a necessary inference, granted the knowledge that only one of the two clauses in the disjunction can be true. The second premise establishes the truth of the disjunction's second clause and that rules out its first clause. So the inference is necessary. Our everyday reasoning depends on knowledge about the premises, which may be perceptions or descriptions. And most of our reasoning is intuitive in the sense that we model only the possibilities in which the fundamental propositions in our premises hold. To model them when they do not hold calls for deliberation—indeed, it is a crucial difference between intuitive and deliberative thinking (this distinction between two sorts of thinking has a long history, starting with Aristotle, and including Pascal, Turing, Wason, and Kahneman). Consider again these two assertions:

It's swollen or it's dilated, but both are not true.

It's swollen or it's not dilated, but both are not true.

We model the two intuitive possibilities in which the first assertion is true:

Swollen

Dilated

And the two intuitive possibilities in which the second assertion is true:

Swollen

Not (dilated)

The possibility of it being swollen is common to both models, and so we judge that the two sentences are both true in this case. The two assertions are therefore consistent with one another. But we err in overlooking what is false in the possibilities. As the earlier analysis shows, when the pupil is swollen, the first assertion implies that it is not dilated, whereas the second assertion implies that it is dilated. So, the two cases of a swollen pupil describe different and incompatible possibilities. The two disjunctions cannot both be true. The inference to the contrary that they can both be true is compelling, but it is an illusion.

The failure to infer proper consequences is a common mistake in the seven earlier test cases. Defenders of cultural relativism were mistaken not to draw the conclusion that the culture of rationalism refuted their doctrine. The proponent of optimists as those who believe optimists exist was mistaken not to infer that it implied that pessimists could be optimists. Ergo, the definition was useless. The proponents of the test for the consistency of beliefs failed to infer that it took to look too long to be plausible for cognition.

Ignorance is no defense in the law: nor should it be in science. Knowledge is essential for reasoning in daily life and in cognitive science. Likewise, ignorance does not excuse any of us for making another broad class of errors—failure to consider scientific principles. They include faults in the design of experiments, such as failure to manipulate a crucial variable as Lubbock did in his study of bees; failure to ensure that participants carry out the required task and allowing them to guess rather than to reason; and failure to allow discoveries outside the scope of a preferred theory and testing only a single sort of logic. Nor does ignorance excuse us from faults in the statistical analysis of results, such as overlooking regression to the mean; the greater departures from reality of small samples than large samples, i.e., the law of large numbers (see Tversky and Kahneman, 1971, who report a study in which experts erred on this point); and ignoring the law of truly large numbers, i.e., with a large enough sample, anything can happen including extraordinary coincidences (Diaconis and Mosteller, 2006; see, e.g., McKay et al., 1999). And ignorance does not excuse us for faults in the interpretation of results, such as the failure to distinguish between a selection of potential evidence that matches a hypothesis and a selection that aims to confirm it. Yet to reiterate an earlier point: there can be useful errors that Turing (1996) and Wilczek (2002) welcomed. So, in mitigation for the neglect of regression to the mean, our ability to create causal explanations is striking and well beyond the competence of any current computer program.

In conclusion, mistakes in thinking are often failures to consider possibilities. They overlook the consequences of hypotheses, facts, and processes. They take for granted choices that violate basic principles of experiments—their design, analysis, and interpretation. Long-standing principles can be wrong. What can possibly happen? If we could always anticipate the actual possibility, our errors in thinking would be far fewer. But no method exists to guarantee correct anticipations. Some neglected possibilities result from mistakes in reasoning, and we now have a method that can help us to do better in our searches for possibilities. A final thanks to Christophe Bernard for the invitation and advice and to two long-term critical readers, Maya Bar-Hillel and Sunny Khemlani, who put me straight on many points.
